# Efficient Asymmetric Reduction of 4-(Trimethylsilyl)-3-Butyn-2-One by *Candida parapsilosis* Cells in an Ionic Liquid-Containing System

**DOI:** 10.1371/journal.pone.0037641

**Published:** 2012-05-25

**Authors:** Bo-Bo Zhang, Wen-Yong Lou, Wen-Jing Chen, Min-Hua Zong

**Affiliations:** 1 State Key Laboratory of Pulp and Paper Engineering, South China University of Technology, Guangzhou, People's Republic of China; 2 Key Laboratory of Industrial Biotechnology, Ministry of Education, School of Biotechnology, Jiangnan University, Wuxi, People's Republic of China; 3 Laboratory of Applied Biocatalysis, South China University of Technology, Guangzhou, People's Republic of China; Queen's University Belfast, United Kingdom

## Abstract

Hydrophilic ionic liquids (ILs) were employed as green solvents to construct an IL-containing co-solvent system for improving the asymmetric reduction of 4-(trimethylsilyl)-3-butyn-2-one by immobilized *Candida parapsilosis* cells. Among 14 hydrophilic ILs examined, 1-(2′-hydroxyl)ethyl-3-methylimidazolium nitrate (C_2_OHMIM·NO_3_) was considered as the most suitable IL for the bioreduction with the fastest initial reaction rate, the highest yield and the highest product *e.e.*, which may be due to the good biocompatibility with the cells. For a better understanding of the bioreduction performed in the C_2_OHMIM·NO_3_-containing co-solvent system, the effects of several crucial variables were systematically investigated. The optimal C_2_OHMIM·NO_3_ content, substrate concentration, buffer pH, co-substrate concentration and temperature were 10% (v/v), 3.0 mmol/L, 5.0, 98.1 mmol/L and 30°C, respectively. Under the optimal conditions, the initial reaction rate, the maximum yield and the product *e.e.* were 17.3 µmol/h g_cell_, 95.2% and >99.9%, respectively, which are much better than the corresponding results previously reported. Moreover, the immobilized cells remained more than 83% of their initial activity even after being used repeatedly for 10 batches in the C_2_OHMIM·NO_3_-containing system, exhibiting excellent operational stability.

## Introduction

Silicon-containing compounds play an important role not only in asymmetric synthesis and functional materials, but also in the preparation of silicon-containing drugs, such as Zifrosilone, Cisobitan and TAC-101{4-[3,5-bis(trimethylsilyl)benzamido] benzoic acid} [1]–[3]. These silicon-containing compounds generally possess greater pharmaceutical activity, higher selectivity and lower toxicity than their carbon counterparts due to the unique physical and chemical characteristics of the silicon atom, such as its larger atomic radius and smaller electronegativity than the carbon atom [4]. Among the useful silicon-containing compounds, enantiopure organosilyl alcohols, which have proved to be versatile intermediates for the synthesis of many chiral pharmaceuticals, agrochemicals, liquid crystals and flavors, have attracted more and more attentions [5], [6]. The enantiopure organosilyl alcohol (*S*)-4-(trimethylsilyl)-3-butyn-2-ol {(*S*)-TMSBOL}, which is a crucial intermediate for the synthesis of 5-lipoxygenase inhibitors, could be prepared by both chemical and biological approaches [7]. Compared to the traditional chemical methods, the economic preparation of enantiopure (*S*)-TMSBOL via asymmetric reduction of the corresponding prochiral ketone 4-(trimethylsilyl)-3-butyn-2-one (TMSB) with whole cell catalysis, has become a subject of great interest due to their high enantioselectivity, mild reaction conditions and low environmental pollution. The major advantages of using whole cells rather than isolated enzymes as the biocatalysts are that cells provide a natural environment for the enzymes, preventing conformational changes in the protein structure that would lead to loss of activity in non-conventional medium, and are able to efficiently regenerate cofactors [8].

To our best knowledge, there is only one study so far related to the asymmetric reduction of TMSB to (*S*)-TMSBOL by an isolated enzyme, in which the yield (78%) and product *e.e.* (57%) were both relatively low [9], possibly due to the low activity and enantioselectivity of the enzyme, as well as the relatively poor efficiency of the biocatalytic system. Recently, we have applied *Candida parapsilosis*, a highly potent carbonyl reductase-producing organism [10], [11], for efficient synthesis of (*S*)-TMSBOL via asymmetric reduction of TMSB in the aqueous system, in terms of the relatively high yield (81.3%) and the excellent product *e.e.* (>99.9%) [12]. Furthermore, an environmental friendly hydrophobic ionic liquid (IL)/buffer biphasic system was successfully used to replace the aqueous system for improving the yield [13]. However, the reaction clearly slowed down when the aqueous system was replaced by the IL/buffer biphasic system (14.5 µmol/h g_cell_ vs 5.5 µmol/h g_cell_), due to the lower availability of the substrate in the aqueous phase and the severe mass transfer limitation. A longer reaction time (around 12 h) was required in the IL-based biphasic system compared to the aqueous system (around 1 h) [13]. Moreover, when applied to large scale production, an IL-based biphasic system often causes detrimental influences such as pronounced emulsification on the biocatalytic process.

Recently, in order to enhance the efficiency of bioreduction process, hydrophilic dialkylimidazolium-based ILs instead of hydrophobic ILs were applied in the whole-cell reaction systems. Hydrophilic dialkylimidazolium-based ILs, which are similar in structure to cationic surfactants, may be able to increase the permeability of microbial cell membrane and then not only lower the product concentration within microbial cells but also reduce the inhibition and toxic effects of the product [14]–[16]. Furthermore, in some cases, hydrophilic ILs can not only improve the enzyme stability but also act as enzyme activators, and result in an enhanced productivity [17]–[21]. Therefore, it seems that hydrophilic ILs are promising and attractive co-solvents for use in the cell-based biocatalytic processes.

**Figure 1:**
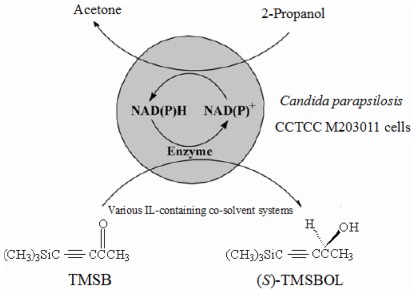
The bioreduction of TMSB to (*S*)-TMSBOL with immobilized *Candida parapsilosis* CCTCC M203011 cells in various IL-containing co-solvent systems.

In this study, for the first time, several hydrophilic dialkylimidazolium-based ILs were used as the co-solvent to improve the bioreduction of TMSB to (*S*)-TMSBOL catalyzed by immobilized *C. parapsilosis* (Figure 1). In this biocatalytic process, TMSB is reduced to enantiopure (*S*)-TMSBOL while converting NAD(P)H to NAD(P)^+^, and the co-substrate 2-propanol is simultaneously oxidized to acetone, presumably driving the reduction reaction by regenerating NAD(P)H from NAD(P)^+^.

## Results and Discussion

### 1. Effect of various hydrophilic ILs on the bioreduction of TMSB to (*S*)-TMSBOL

Until now, a number of reports have been carried out on biocatalytic reduction of ketones in various IL-containing reaction systems, in which the performances of biocatalysts were closely related to the kinds and combination of cation and anion of ILs, and the effect of different ILs on the biocatalytic reactions has been found to vary greatly [20], [22]–[25]. Hence, in order to explore the effect of ILs with different combination of cation and anion, the bioreduction of TMSB to (*S*)-TMSBOL with immobilized *C. parapsilosis* cells were conducted in various hydrophilic IL-containing co-solvent systems.

As shown in Table 1, the biocatalytic reduction of TMSB exhibit great difference with the use of different ILs. Surprisingly, in the BF_4_^−^-based IL-containing co-solvent systems, the substrate TMSB disappeared completely after reaction for 1 h without any desired product TMSBOL found in the reaction medium. The unexpected phenomenon may be attributable to the similar phenomenon found in aqueous buffer system, in which the substrate TMSB was unstable and may undertake a non-biocatalytic side reaction in some cases [12]. A further investigation on the underlying mechanism by which BF_4_^−^-based IL affects the bioreduction of TMSB is required and now is underway in our laboratory. Except with the BF_4_^−^-based ILs, the *C. parapsilosis* cells were capable of catalyzing the asymmetric reduction of TMSB in the other IL-containing co-solvent systems with a high product *e.e.* of above 99.9%. In the case of the C_2_OHMIM^+^-based ILs (C_2_OHMIM·PF_6_ and C_2_OHMIM·NO_3_), the anions significantly influenced the initial reaction rate and the maximum yield of the bioreduction (Table 1, entries 7, 8). For C_n_MIM·NO_3_ (n = 2, 4) and C_n_MIM·Br (n = 4–7), the initial reaction rate and the maximum yield obviously decreased with the elongation of the alkyl chain attached to the cation (i.e. increasing n value) (Table 1, entries 9–10, 12–15). These results were consistent with other previous studies, in which similar relationships between increasing length of the alkyl chain of the imidazolium cation and the increasing toxicity of the ionic liquid to microorganisms have been clearly exhibited [20], [26]–[28]. Among the 14 hydrophilic ILs examined, C_2_OHMIM·NO_3_ was considered as the most suitable IL with the fastest initial reaction rate and the highest yield, and accordingly used in the subsequent experiments.

**Table 1:** Effect of various hydrophilic ILs on the bioreduction of TMSB with immobilized *Candida parapsilosis* cells.

Entries	Media	Initial reaction rate (µmol/h g_cell_)	Yield (%)	*e.e.* (%)
1	Aqueous buffer	5.5	81.3	>99.9^a^
2	C_2_MIM·BF_4_-buffer	n.d.^b^	n.d.	n.d.
3	C_3_MIM·BF_4_-buffer	n.d.	n.d.	n.d.
4	C_4_MIM·BF_4_-buffer	n.d.	n.d.	n.d.
5	C_5_MIM·BF_4_-buffer	n.d.	n.d.	n.d.
6	C_2_OHMIM·BF_4_-buffer	n.d.	n.d.	n.d.
7	C_2_OHMIM·PF_6_-buffer	13.1	64.7	>99.9
8	C_2_OHMIM·NO_3_-buffer	14.8	79.2	>99.9
9	C_2_MIM·NO_3_-buffer	11.1	55.2	>99.9
10	C_4_MIM·NO_3_-buffer	8.9	47.5	>99.9
11	C_4_MIM·Cl-buffer	6.5	33.7	>99.9
12	C_4_MIM·Br-buffer	14.0	73.7	>99.9
13	C_5_MIM·Br-buffer	13.6	71.1	>99.9
14	C_6_MIM·Br-buffer	12.3	68.1	>99.9
15	C_7_MIM·Br-buffer	6.1	53.3	>99.9

a Data taken from Zhang et al. [12];

b n.d. = Not detected

Reaction conditions: 3.0 mmol/L TMSB, 4.0 mL TEA-HCl buffer (100 mmol/L, pH 5.0) containing 15% (v/v) different kinds of ILs, 65.3 mmol/L 2-propanol, and 0.15 g/mL immobilized *C. parapsilosis* cells, 30°C, 180 r/min.

### 2. Cell viability of *C. parapsilosis* in various hydrophilic IL-containing co-solvent systems

Recent reports indicate that the ILs can exert great impact on the cell viability, and hence biocompatibility of an IL must be established prior to its application in a whole-cell biocatalytic process [29]–[31]. Moreover, it is well known that most ketone substrates and/or their alcohol reduction products exhibit pronounced toxicity to cells [32], [33]. Accordingly, cell viability of *C. parapsilosis* was examined in various hydrophilic IL-containing co-solvent systems, with and without the addition of substrate.

**Figure 2:**
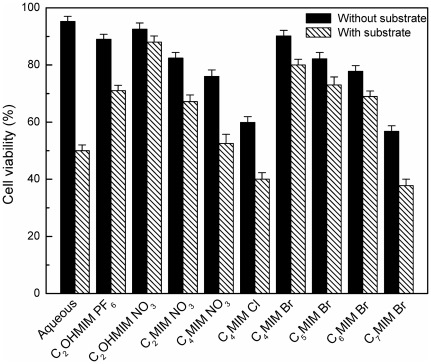
Cell viability of *Candida parapsilosis*. Conditions: Cells were exposed to co-solvent systems comprising 10% (v/v) of various ILs and TEA-HCl buffer (100 mmol/L, pH 5.0), with and without substrate (3.0 mmol/L TMSB).

As shown in Figure 2, without the addition of substrate, the cell viability was lower in all the IL-containing systems than in aqueous buffer, suggesting that the ILs were toxic to the cells to some extent. Among all the tested ILs, C_2_OHMIM·NO_3_ displayed the best biocompatibility with the cells.

On the other hand, with the addition of substrate, the cell viability decreased obviously in all the reaction systems as compared with that in the absence of substrate, indicating that the substrate and/or product had substantial toxicity to the cells. Moreover, all ILs induced different degrees of effect on cell viability. Notably, the cells lost about 100% of their catalytic activity quickly in the BF_4_^−^-based IL-containing systems in the presence of substrate, indicating that these BF_4_^−^-based ILs had very low biocompatibility with and substantial toxicity to the cells (data not shown). This observation could also partially explain the poor biotransformation results achieved in these reaction media (Table 1). In addition, the cell viability clearly decreased with the elongation of the alkyl chain attached to the cation in C_n_MIM·NO_3_ (n = 2, 4) and C_n_MIM·Br (n = 4–7), which were in accordance with the observed decrease in initial reaction rate and maximum yield with increasing length of the alkyl chain in the IL cation (Table 1).

Among all the tested systems (including the aqueous buffer system used as the control), the highest cell viability (88%) with the addition of substrate was observed in the C_2_OHMIM·NO_3_-containing co-solvent system. This was consistent with the fastest initial reaction rate, the highest yield and product *e.e.*, which were also achieved in the C_2_OHMIM·NO_3_-based system (Table 1). Moreover, the fall in cell viability due to the addition of substrate was only 5% in the C_2_OHMIM·NO_3_-containing co-solvent medium, which was much less than the fall in viability (around 45%) when the substrate is added to the aqueous buffer system. Obviously, the distinct effects of hydrophilic ILs on the bioreduction could be attributed to the cell viability in the various hydrophilic IL-containing co-solvent systems.

### 3. Optimization of the reduction of TMSB in the C_2_OHMIM·NO_3_-containing co-solvent system

For a better understanding of the bioreduction performed in the co-solvent system containing hydrophilic IL C_2_OHMIM·NO_3_, the effects of several crucial variables such as IL content, substrate concentration, buffer pH, co-substrate concentration and temperature were systematically studied.

#### 3.1 Effect of IL content

**Figure 3:**
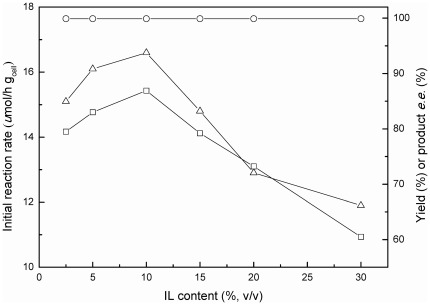
Effect of C_2_OHMIM·NO_3_ content on the bioreduction of TMSB. Symbols: (△) the initial reaction rate; (□) the maximum product yield; (○) the product *e.e.* All products have the (*S*) configuration. Reaction conditions: 3.0 mmol/L TMSB, 4.0 mL TEA-HCl buffer (100 mmol/L, pH 5.0) containing different amount of C_2_OHMIM·NO_3_, 65.3 mmol/L 2-propanol, and 0.15 g/mL immobilized *C. parapsilosis* cells, 30°C, 180 r/min.

Although use of hydrophilic IL could improve enzyme activity and stability, as well as reduced substrate surplus and product inhibitions, the content of hydrophilic IL in the co-solvent system should also be carefully controlled to avoid inactivation of enzyme at high IL content [18], [22], [34]. As shown in Figure 3, the content of C_2_OHMIM·NO_3_ had great influence on the bioreduction of TMSB with immobilized *C. parapsilosis* cells. Both the initial reaction rate and the maximum yield increased with the rise of IL content from 2.5% (v/v) to 10% (v/v). However, further increase in the IL content resulted in a sharp decline in the initial reaction rate and the maximum yield, suggesting that too high concentration of C_2_OHMIM·NO_3_ caused not only a high ionic strength of the reaction medium that might inactivate the cells or the enzymes present in the cells, but also a high viscosity of the reaction mixture, which can limit the diffusion of substrates and products in and out of the immobilized cells. Within the tested IL content range between 2.5% (v/v) and 30% (v/v), the product *e.e.* was consistently above 99.9%, indicating a great enantioselectivity of the enzyme within the immobilized cells. And obviously, the optimal content of C_2_OHMIM·NO_3_ in the co-solvent system was 10% (v/v).

#### 3.2 Effect of substrate concentration

As shown in Table 2, the initial reaction rate and the maximum yield increased with increasing substrate concentration up to 3.0 mmol/L, beyond which further increase in substrate concentration led to a clear drop in the initial reaction rate and the maximum product yield, possibly owing to the substrate inhibition at high concentrations of substrate. Throughout the tested range of substrate concentrations, the product *e.e.* showed no variation and kept above 99.9%. Therefore, the suitable substrate concentration for the bioreduction carried out in the C_2_OHMIM·NO_3_-containing co-solvent system was 3.0 mmol/L, with respect to the initial reaction rate, the maximum yield and the product *e.e*.

**Table 2:** Effect of substrate concentration on the bioreduction of TMSB with immobilized *Candida parapsilosis* cells.

Substrate concentration (mmol/L)	Initial reaction rate (µmol/h g_cell_)	Yield (%)	*e.e.* (%)
1.5	12.6	86.8	>99.9
3.0	16.6	86.9	>99.9
4.5	15.8	75.1	>99.9
6.0	12.8	66.1	>99.9
9.0	12.3	62.5	>99.9
12.0	9.0	54.1	>99.9

Reaction conditions: various concentrations of TMSB, 4.0 mL TEA-HCl buffer (100 mmol/L, pH 5.0) containing 10% (v/v) C_2_OHMIM·NO_3_, 65.3 mmol/L 2-propanol, and 0.15 g/mL immobilized *C. parapsilosis* cells, 30°C, 180 r/min.

#### 3.3 Effect of buffer pH

**Figure 4:**
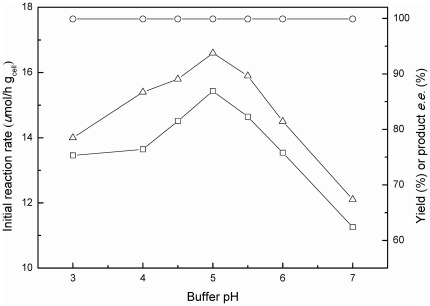
Effect of buffer pH on the bioreduction of TMSB. Symbols: (△) the initial reaction rate; (□) the maximum product yield; (○) the product *e.e.* All products have the (*S*) configuration. Reaction conditions: 3.0 mmol/L TMSB, 4.0 mL TEA-HCl buffer (100 mmol/L, various pH) containing 10% (v/v) C_2_OHMIM·NO_3_, 65.3 mmol/L 2-propanol, and 0.15 g/mL immobilized *C. parapsilosis* cells, 30°C, 180 r/min.

Buffer pH plays an important role on the ionic state of substrate and enzymes, as well as the enantioselectivity, activity and stability of the enzymes involved in the bioreduction, thus influencing the reaction rate, product yield and *e.e.* value to a great extent [35], [36]. Figure 4 depicts the significant effect of buffer pH on the bioreduction in the C_2_OHMIM·NO_3_-containing co-solvent system. The reaction markedly accelerated and the maximum product yield enhanced with increasing buffer pH from pH 3.0 to 5.0. Further increase in buffer pH resulted in a sharp decline in the initial reaction rate and the maximum yield. Additionally, the buffer pH showed little impact on the product *e.e.*, which remained above 99.9% within the range examined, indicating that there was no problem with activity of undesired isoenzymes within this range of pH. Hence, it is clear that pH 5.0 is the optimal buffer pH for the reaction.

#### 3.4 Effect of co-substrate concentration

With the use of whole cells, bioreduction reactions can perform effectively without extra addition of the expensive coenzyme and only a co-substrate is necessary for recycling the coenzyme within the cells [8], [37], [38]. 2-Propanol, which has been found to be a suitable co-substrate for coenzyme regeneration in whole cells [39], was used as in this reaction and the effect of its concentration was investigated. The great impact of co-substrate concentration on the bioreduction was exhibited in Table 3. Increasing concentration of 2-propanol from 32.7 mmol/L to 98.1 mmol/L gave rise to an increase in the initial reaction rate from 14.4 µmol/h g_cell_ to 17.3 µmol/h g_cell_, while the yield markedly increased from 75.4% to 95.2%. However, further increase in the concentration of 2-propanol led to poor reaction rate and the maximum product yield, possibly because of the negative effects of the excessive 2-propanol on the cell viability [13]. The concentration of 2-propanol showed little influence on the product *e.e.*, which kept above 99.9% within the range tested. Thus, the most suitable concentration of 2-propanol for the reaction was 98.1 mmol/L.

**Table 3:** Effect of co-substrate concentration on the bioreduction of TMSB with immobilized *Candida parapsilosis* cells.

Concentration of 2-propanol (mmol/L)	Initial reaction rate (µmol/h g_cell_)	Yield (%)	*e.e.* (%)
32.7	14.4	75.4	>99.9
65.3	16.6	86.9	>99.9
98.1	17.3	95.2	>99.9
130.6	16.5	84.8	>99.9
163.5	15.0	74.5	>99.9
195.9	12.3	64.3	>99.9

Reaction conditions: 3.0 mmol/L TMSB, 4.0 mL TEA-HCl buffer (100 mmol/L, pH 5.0) containing 10% (v/v) C_2_OHMIM·NO_3_, various concentrations of 2-propanol, and 0.15 g/mL immobilized *C. parapsilosis* cells, 30°C, 180 r/min.

#### 3.5 Effect of reaction temperature

Reaction temperature is another important parameter for a biocatalysis process, especially for the bioreduction with whole cells, in which the isoenzymes might be activated at different reaction temperatures [8], [40].

**Figure 5:**
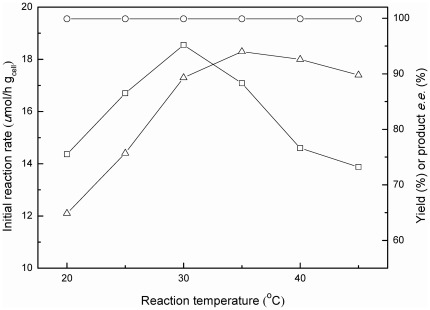
Effect of reaction temperature on the bioreduction of TMSB. Symbols: (△) the initial reaction rate; (□) the maximum product yield; (○) the product *e.e.* All products have the (*S*) configuration. Reaction conditions: 3.0 mmol/L TMSB, 4.0 mL TEA-HCl buffer (100 mmol/L, pH 5.0) containing 10% (v/v) C_2_OHMIM·NO_3_, 98.1 mmol/L 2-propanol, and 0.15 g/mL immobilized *C. parapsilosis* cells, various reaction temperatures, 180 r/min.

As illustrated in Figure 5, a rise in reaction temperature markedly boosted the initial reaction rate up to 35°C, and further increase in temperature led to an obvious decline in the initial reaction rate, while the product *e.e.* showed little variation. When the temperature was below 30°C, higher product yield was achieved along with the increase of reaction temperature. The maximum yield sharply decreased when the temperature was above 30°C, which might be due to the partial inactivation of the cells at a higher temperature. Taking into consideration of the initial reaction rate, the yield and the product *e.e.*, 30°C appeared to be the appropriate reaction temperature for the reaction.

Under the optimized conditions described above, the initial reaction rate in the C_2_OHMIM·NO_3_-containing co-solvent system reached 17.3 µmol/h g_cell_, which was much faster than that in both aqueous monophasic system (14.5 µmol/h g_cell_) [12] and the C_4_MIM·PF_6_-based IL/buffer biphasic system (5.5 µmol/h g_cell_) [13]. Accordingly, the reaction time was greatly shortened from about 12 h to 1 h when replaced the IL/buffer biphasic system to the C_2_OHMIM·NO_3_-containing co-solvent system. Moreover, the maximum product yield in the C_2_OHMIM·NO_3_-containing system (95.2%) was not significant different with that of the C_4_MIM·PF_6_/buffer biphasic system (97.7%) [13] but much higher than that of the aqueous system (81.3%) [12]. The product *e.e.* was kept above 99.9% at the three different reaction media. Obviously, the bioreduction of TMSB with immobilized *C. parapsilosis* cells proceed more efficiently in the C_2_OHMIM·NO_3_-containing co-solvent system than in the other reaction systems.

### 4. Operational stability of immobilized *C. parapsilosis* cells

**Figure 6:**
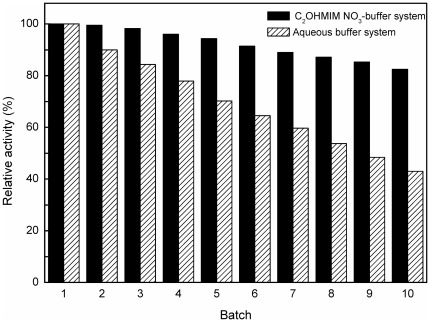
Operational stability of immobilized *Candida parapsilosis* cells. Reaction conditions with C_2_OHMIM·NO_3_-buffer system: 3.0 mmol/L TMSB, 4.0 mL TEA-HCl buffer (100 mmol/L, pH 5.0) containing 10% (v/v) C_2_OHMIM·NO_3_, 98.1 mmol/L 2-propanol, and 0.15 g/mL immobilized *C. parapsilosis* cells, 30°C, 180 r/min, 1 h per batch; Reaction conditions with aqueous buffer system: 3 mmol/L TMSB, 4.0 mL of TEA-HCl buffer (100 mmol/L, pH 5.0), 65.3 mmol/L 2-propanol, 0.15 g/mL immobilized *C. parapsilosis* cells, 30°C, 180 r/min, 1 h per batch. The relative activity of the immobilized cells in the first batch was defined as 100%.

To obtain a deeper understanding on the effect of IL on the whole cell bioctalytic processes, it is necessary to make a comparative investigation on the operational stability of the immobilized *C. parapsilosis* cells in the C_2_OHMIM·NO_3_-buffer system and aqueous buffer system. As shown in Figure 6, the immobilized cells still remained above 83% of their original activity even after being used repeatedly for 10 batches in the C_2_OHMIM·NO_3_-containing co-solvent system and the results were much better than that in the aqueous system (43%) after the same operational batches. The operational stability in this IL-containing monophasic system was also comparable to that in the C_4_MIM·PF_6_/buffer biphasic system (85%) [13]. Obviously, the cells had great operational stability in the C_2_OHMIM·NO_3_-containing system. The excellent solvent properties and the good biocompatibility of the IL C_2_OHMIM·NO_3_ for the microbial cells could partly account for the observations. The improved interactions between the IL and the carrier (calcium alginate) [41] used for the immobilization of *C. parapsilosis* cells may also contribute to the good operational stability of the immobilized cells in C_2_OHMIM·NO_3_-containing system.

### 5. Conclusions

The hydrophilic IL C_2_OHMIM·NO_3_ can markedly enhance the efficiency of TMSB reduction to enantiopure (*S*)-TMSBOL with immobilized *C. parapsilosis* cells. In addition, the immobilized cells exhibit excellent operational stability in the C_2_OHMIM·NO_3_-containing co-solvent system, which may be due to the excellent solvent properties and the good biocompatibility of the IL C_2_OHMIM·NO_3_. More insights into the underlying mechanism of hydrophilic ILs on the bioreduction process are required and now are underway in our laboratory.

## Materials and Methods

### 1. Biological and chemical materials

*Candida parapsilosis* CCTCC M203011 from the China Center for Type Culture Collection (CCTCC, Wuhan, China) [11] was kindly donated by Professor Yan Xu (Key Laboratory of Industrial Biotechnology, Ministry of Education, School of Biotechnology, Jiangnan University, China).

4-(Trimethylsilyl)-3-butyn-2-one (TMSB, 97% purity), 4-(trimethylsilyl)-3-butyn-2-ol (TMSBOL, 97% purity) and n-decane (>99% purity) were purchased from Sigma-Aldrich (USA). The seven ILs used in this work, 1-(2′-hydroxyl)ethyl-3-methylimidazolium nitrate (C_2_OHMIM·NO_3_), 1-(2′-hydroxyl)ethyl-3-methylimidazolium hexafluorophosphate (C_2_OHMIM·PF_6_), 1-(2′-hydroxyl)ethyl-3-methylimidazolium tetrafluoroborate (C_2_OHMIM·BF_4_), 1-ethyl-3-methylimidazolium nitrate (C_2_MIM·NO_3_), 1-butyl-3-methylimidazolium nitrate (C_4_MIM·NO_3_), 1-ethyl-3-methylimidazolium tetrafluoroborate (C_2_MIM·BF_4_) and 1-butyl-3-methylimidazolium tetrafluoroborate (C_4_MIM·BF_4_) were purchased from Lanzhou Institute of Chemical Physics (China) and were all of over 99% purity. The other seven ILs, 1-propyl-3-methylimidazolium tetrafluoroborate (C_3_MIM·BF_4_), 1-pentyl-3-methylimidazolium tetrafluoroborate (C_5_MIM·BF_4_), 1-butyl-3-methylimidazolium chloride (C_4_MIM·Cl), 1-butyl-3-methylimidazolium bromide (C_4_MIM·Br), 1-pentyl-3-methylimidazolium bromide (C_5_MIM·Br), 1-hexyl-3-methylimidazolium bromide (C_6_MIM·Br) and 1-heptyl-3-methylimidazolium bromide (C_7_MIM·Br) were kindly donated by Professor Xue-Hui Li (Department of Chemical Engineering, South China University of Technology, China) and were all of above 96% purity. All other chemicals were obtained from commercial sources and were of analytical grade.

### 2. Cultivation and immobilization of *C. parapsilosis* cells

*C. parapsilosis* cells were cultivated and immobilized according to our previously described methods [12].

### 3. General procedure for the bioreduction of TMSB to (*S*)-TMSBOL in IL-containing systems

In a typical experiment, 4.0 ml of different IL-containing co-solvent systems or aqueous TEA-HCl buffer (100 mmol/L) monophasic system were contained in a 20 mL Erlenmeyer flask capped with a septum. Alginate beads were loaded with 31% (w/w) *C. parapsilosis* cells {based on cell wet mass (cwm)} and 0.15 g of these cell-loaded alginate beads were added per mL of the solvent, together with a predetermined quantity of co-substrate (2-propanol). The reaction mixture was pre-incubated for 15 min at the appropriate temperature and shaking rate. Then, the reactions were initiated by adding TMSB at a predetermined concentration. Aliquots (10 µL) were withdrawn at specified time intervals from the co-solvent system, and then the product as well as the residual substrate was extracted with n-hexane (50 µL) containing 5.1 mmol/L n-decane (as an internal standard), prior to GC analysis. Initial reaction rate is defined as the initial rate of the product formation in the total reaction system, expressed as the specific activity in µmol product per hour per gram of cell. Details of the IL content, substrate concentration, buffer pH, co-substrate concentration, reaction temperature and shaking rate are specified for each case.

### 4. Cell viability assay

The viability of *C. parapsilosis* cells was assayed after incubating the alginate-immobilized cells for 6 h in various co-solvent systems consisting of hydrophilic ILs (15%, v/v) and TEA-HCl buffer (100 mmol/L, pH 5.0) in the absence of substrate and in the presence of substrate (3.0 mmol/L TMSB), respectively. The beads of immobilized *C. parapsilosis* cells were withdrawn from the reaction systems and then added to 0.1 M trisodium citrate to dissolve the beads. The microbial cell suspension was diluted and dyed with 0.1% Methylene Blue for 5 min. Micrographs were taken and analyzed for blue dead cells and colourless viable ones. The cell viability was expressed as the percentage of viable ones in the total cells and the values were given as mean value ± standard deviation (n = 3).

### 5. GC analysis

Reaction mixtures were analyzed according to the GC analysis method in our previous work [12]. The retention times for TMSB, n-decane and TMSBOL were 5.1, 5.7, and 10.5 min, respectively. Moreover, the product configuration was confirmed to be (*S*)-TMSBOL with the method in our previous work [12]. The average error for this determination was less than 1.0%. All reported data were averages of experiments performed at least in duplicate.

### 6. Operational stability of immobilized *C. parapsilosis* cells

In order to assess the operational stability of the cells, the re-use of the immobilized *C. parapsilosis* cells was investigated in both the C_2_OHMIM·NO_3_-buffer co-solvent system and the aqueous buffer system. Initially, 0.6 g immobilized *C. parapsilosis* cells were added into separate screw-capped vials each containing 4.0 mL of the appropriate medium (C_2_OHMIM·NO_3_-buffer system or TEA-HCl buffer system). Then, the bioreduction was carried out at the optimal conditions in the various media and was repeated over 10 batches without changing the immobilized cells. Between batches, the immobilized cells were filtered off from the reaction mixture, washed twice with distilled water, and added to a fresh batch of reaction medium. The reduction activity of the cells was assayed in each batch. The relative activity of the cells employed for the first batch was defined as 100%.
